# Beyond Myiasis: Understanding Environmental Factors, Maggots, and Infection Risks in Xylazine-Associated Wounds

**DOI:** 10.7759/cureus.87518

**Published:** 2025-07-08

**Authors:** Alexander H Chang, Kenny Oh, Sameer Patel, Adam Walchak

**Affiliations:** 1 Division of Plastic and Reconstructive Surgery, Lewis Katz School of Medicine (LKSOM) Temple University Hospital/Fox Chase Cancer Center, Philadelphia, USA; 2 Department of Vascular Surgery, Lewis Katz School of Medicine (LKSOM) Temple University Hospital, Philadelphia, USA

**Keywords:** ignatzschineria, ignatzschineria bacteremia, larval therapy, maggot debridement, maggot therapy, myiasis, wound care, xylazine-associated wounds

## Abstract

The emergence of xylazine-associated wounds, highlighted by case reports from Philadelphia in 2022, has been linked to the use of this veterinary sedative as an adulterant in the unregulated drug supply and has brought renewed attention to the challenges of complex wound management, particularly in vulnerable populations. Among these challenges are unusual infectious complications, including cases of larva-associated infections such as *Ignatzschineria* bacteremia in maggot-colonized wounds, raising critical questions about the role of environmental exposure, wound neglect, and the presence of maggots. Clinicians must recognize that rare infections often arise not from the drug itself but from environmental exposure and delayed wound care. Furthermore, it is essential to distinguish between uncontrolled myiasis (spontaneous, unsanitary maggot infestation) and the therapeutic application of sterile *Lucilia sericata* larvae through medically supervised larval therapy. Building on historical observations and contemporary wound care literature, we explore how larval therapy offers a precise, cost-effective method for wound debridement, particularly valuable in situations in which definitive surgical care is delayed or unfeasible. As clinicians confront the rising burden of xylazine-related wounds, a nuanced understanding of environmental infectious risks and biosurgical interventions can expand the surgeon's wound care armamentarium and improve outcomes not only for patients with drug-induced wounds but also for individuals with chronic wounds.

## Editorial

The rise of xylazine-associated wounds has brought renewed attention to the complexity of wound pathology. These wounds represent a convergence of pharmacologic injury, behavioral health challenges, and environmental exposure, often resulting in extensive tissue necrosis, impaired healing, and infectious complications. Rare infections such as *Ignatzschineria* bacteremia have recently been reported in maggot-colonized wounds, highlighting the intersection between wound neglect, environmental contamination, and evolving wound microbiology [1]. Such infections have been documented previously in non-drug-related cases, such as those complicated by diabetes, peripheral vascular disease, and neglected soft tissue injuries. These associations suggest that environmental exposure, wound neglect, and delayed care are the principal factors leading to the occurrence of such infections, rather than any intrinsic property of xylazine itself.

The variety of pathogens available in the environment may play a more significant role in determining the type of infection than the direct tissue injury caused by the drug injection. To illustrate this point, an individual with a chronic xylazine-induced wound exposed to contaminated waters by a wharf may be at risk for *Shewanella* bacteremia [2]. Similarly, zoonotic involvement, such as handling a stray cat, could lead to *Erysipelothrix rhusiopathiae* bacteremia, endocarditis, or even hemotropic mycoplasmosis (formerly eperythrozoonosis) [3,4]. In warmer climates such as Florida, where cases of xylazine-associated wounds have been documented, it is medically feasible for a patient to develop mycetoma (Madura foot) [5-7]. Documented clinical cases illustrate that environmental conditions significantly influence the types of infections that develop in wounds, regardless of how rare or unusual the pathogen may be. Clinicians should maintain a broad infectious differential diagnosis when evaluating chronic wounds, particularly in patients with environmental exposure risks, such as those experiencing homelessness, animal contact, or inadequate access to timely wound care.

The presence of maggots and subsequent *Ignatzschineria* infection in chronic wounds has been documented in the context of unsanitary conditions (e.g., homelessness) and prolonged neglect (e.g., chronic alcoholism) [8]. These cases underscore the significant role of environmental exposure in the development of wound pathology. However, it is important to recognize that not all maggots are harmful. Under controlled conditions, maggots have long offered a biologically selective method of wound debridement, predating the advent of modern antibiotics. Appreciating this distinction requires revisiting the remarkable history of medicinal maggots in clinical practice.

From the keen observations of the French barber-surgeon Ambroise Paré, who noted in the 16th century that maggot-infested wounds often healed better, to those of Dominique Jean Larrey, Napoleon’s chief surgeon, military physicians throughout history have noted the beneficial presence of maggots [9]. Despite such anecdotal observations, maggot therapy was not formally studied until the late 1920s, when William S. Baer, drawing inspiration from historical military surgeons, began developing sterile maggot therapy for chronic osteomyelitis and non-healing wounds, which he described in his seminal paper [10].

Larval therapy harnesses the selective biological debridement properties of the larvae of *Lucilia sericata*, the green bottle fly [11]. As early as 1935, the antimicrobial properties of maggot secretions and excretions were scientifically studied, demonstrating their potent antimicrobial effects against major wound pathogens [12]. While maggots are often seen as mere vectors in unsanitary environments, it is worth considering that they may also serve a protective role by debriding necrotic tissue and reducing bacterial load. More recent studies have shown that a considerable number of bacteria die as they pass through the digestive tract of maggots [13]. One study found maggot secretions shift monocyte-macrophage differentiation away from a pro-inflammatory state toward a pro-angiogenic type, which may contribute to improved wound healing by reducing excessive inflammation and promoting tissue regeneration [14]. It is plausible that, even in unsanitary wounds, maggots may slow the progression of deeper tissue necrosis despite the presence of bacteremia. Thus, the distinction between unregulated myiasis in neglected wounds and clinically controlled larval therapy is crucial, as both patients and healthcare professionals may have an inherent aversion to such a practice.

Establishing a formal case series documenting the successful application of larval therapy in xylazine-associated wounds, including cases involving pre-existing maggot colonization, could provide critical clinical evidence to support the broader adoption of biosurgical debridement strategies. Such a series would not only validate the therapeutic potential of sterile *Lucilia sericata* larvae in this emerging patient population but also help delineate best practices for the safe, effective integration of larval therapy into complex wound management protocols. In a healthcare landscape increasingly burdened by patients whose chronic wounds are exacerbated by factors such as substance use, addiction, unstable housing, and prolonged exposure to unhygienic environments, documenting these cases could further clarify the role of larval therapy as a biologically selective, cost-effective adjunct-uniquely positioned to serve patients for whom traditional wound care pathways are delayed, inaccessible, or ineffective.

**Figure 1:**
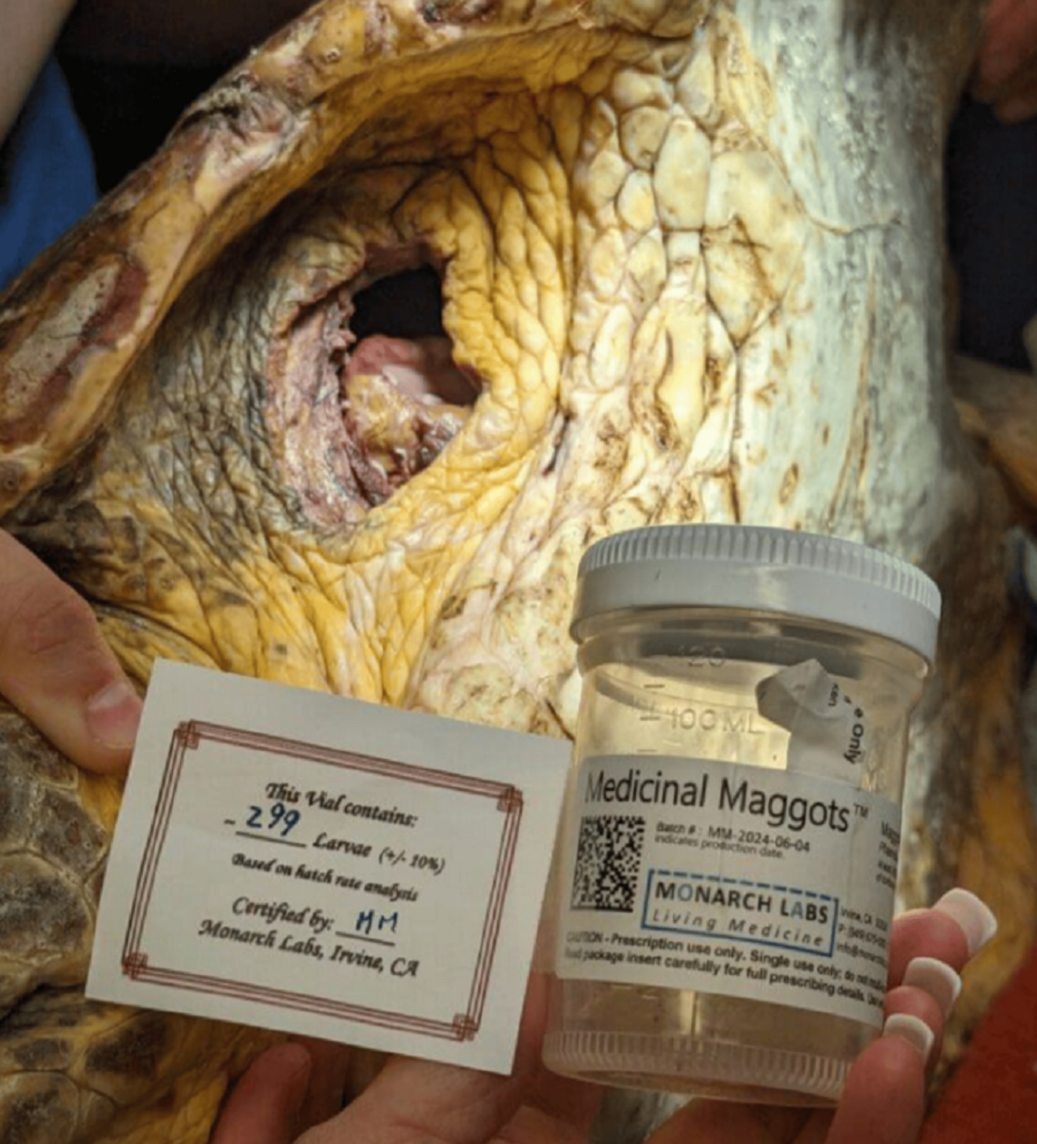
Medicinal Maggots Sterile *Lucilia sericata* larvae from Monarch Labs prepared for biosurgical debridement of a wound for a sea turtle at Brevard Zoo. Larval therapy offers a versatile, targeted approach to wound debridement across human and veterinary settings, particularly in cases where surgical options are limited or delayed. Image used with permission from Monarch Labs [16].

Unlike traditional surgical interventions, larval therapy can be administered without the need for an operating room, surgical instruments, or highly specialized personnel, making it especially valuable in resource-limited settings or for patients for whom serial operations, prolonged hospitalization, or reconstruction are delayed or unfeasible. Maggot debridement's ability to stabilize wounds, reduce bacterial bioburden, and promote granulation tissue offers a necessary bridge for patients undergoing addiction treatment or withdrawal who are not yet candidates for definitive surgical repair, such as free flap closure [15]. As such, larval therapy is not merely a historical curiosity but a practical, evidence-supported option with significant implications for public health. So versatile are maggots that their therapeutic potential extends beyond human medicine; larval therapy has also been explored and utilized in veterinary medicine and wildlife rehabilitation (Figure 1).

By recognizing and embracing the biosurgical capabilities of our entomological allies, the medical community has an opportunity to foster greater interdisciplinary collaboration across fields, including surgery, infectious diseases, addiction medicine, and wound care. Clinicians can reframe misconceptions surrounding both xylazine-associated wounds and larval presence. Infections like *Ignatzschineria* do not stem from xylazine injection itself, but from environmental exposure and delayed wound care. Conflating myiasis with therapeutic maggots risks dismissing a safe, effective, and biologically selective method of debridement. Building clinician and institutional familiarity with larval therapy could also drive innovation in outpatient wound care, reduce hospital resource utilization, and expand access to care for marginalized populations who are historically underserved by traditional surgical pathways. Embracing maggots and their therapeutic potential stands to enhance the wound care armamentarium, expanding therapeutic options for both marginalized patient populations, such as individuals affected by substance use, and the broader population of patients with chronic, non-healing wounds.
